# Pandemic Risk Management for Public Health Care Schemes

**DOI:** 10.3389/fpubh.2021.700021

**Published:** 2021-07-27

**Authors:** Zhengqiao Liu, Yang-Che Wu, Mei-Chih Wang, Yue Ding, Tien-Trung Nguyen

**Affiliations:** ^1^Business School of Yango University, Fuzhou, China; ^2^Department of Finance, College of Finance, Feng Chia University, Taichung, Taiwan; ^3^Center for Chinese Social and Management Studies, Tunghai University, Taichung, Taiwan; ^4^College of Finance and Statistics, Hunan University, Changsha, China; ^5^Faculty of Finance and Accounting, Nguyen Tat Thanh University, Ho Chi Minh City, Vietnam

**Keywords:** pandemic risk management, COVID-19, pandemic probability model, public health care scheme, pandemic hedging

## Abstract

The coronavirus disease 2019 (COVID-19) caused by a novel coronavirus, severe acute respiratory syndrome coronavirus 2, has caused a large death, a range of serious health problems, and significant economic costs in many countries around the world. This study analyzes statistical characteristics of pandemic disasters using historical records since the Middle Ages. Compared to literature which studies the effect of the COVID- 19 pandemic on the financial market, this paper attempts to find two financial instruments in the financial market to hedge pandemic risks. Two instruments could be useful for public health care schemes to increase their assets or decrease their liabilities during the pandemic period, namely, assets in the form of a biotechnology investment portfolio and liabilities in the form of pandemic bonds. Empirical results show the feasibility of such instruments and the informational efficiency of the U.S. stock market.

## Introduction

Epidemics and pandemics have afflicted humanity throughout recorded history, as shown in Table 1. An epidemic is defined as the rapid spread of disease in a large group of individuals beyond the normal level of infection that would be expected in a given population and region over a short period of time. A pandemic refers to an epidemic that has spread over several countries or continents. Compared with sudden catastrophic events (earthquakes, floods, hurricanes, and volcanic eruptions), epidemics and pandemics occur over a relatively long period, as there can be many months or even years between the beginning and the end of these health disasters. For example, to date, the current coronavirus disease 2019 (COVID-19) outbreak has lasted over a year. Therefore, there would be enough time to make use of financial instruments to hedge pandemic risk.

**Table 1:** Pandemics since the middle ages.

**Years**	**Diseases**	**Reported region**	**Deaths**
1347–1351	Bubonic Plague	Europe	Over 50 million
1489	Typhus	Europe	17,000
1500s	Smallpox	Europe, Americas	12–25 million in Mexico
1510	Influenza	Global	Unknown
1557–1558	Influenza	Europe	Unknown, 8,000 in London
1580	Influenza	Asia, Africa, Europe, America	Unknown, 8,000 deaths in Rome
1635, 1656	Measles	Britain, Europe	Unknown
1700s	Yellow Fever	Americas, Europe, Africa	10,000 deaths in Philadelphia
1729–1730, 1732–1733,	Influenza	Global	0.5 million
1761–1762	Influenza	Americas, Europe	Unknown
1780–1782	Influenza	China, India, Europe, America	Unknown
1788–1790	Influenza	Europe, North America	0.8 million
1800s	Cholera	Europe, Americas, Asia	50–100 million
1830–1831, 1832–1833	Influenza	China, Russia, Europe	0.9 million
1889–1893	Influenza/Russian Flu	Global	1 million
1918–1919	Influenza/Spanish Flu	Global	40–50 million
1957–1958	Influenza/Asian Flu	Global	1–1.5 million
1968	Influenza/Hong Kong	Global	0.75–1 million
1979	HIV/AIDS	Global	35 million
1977–1978	Influenza/Russian Flu	Global	Unknown
2009–2010	Influenza/Swine Flu	Global	284,500
2003	SARS	Global	774
2019	COVID-19	Global	2.9 million

**Figure 1:**
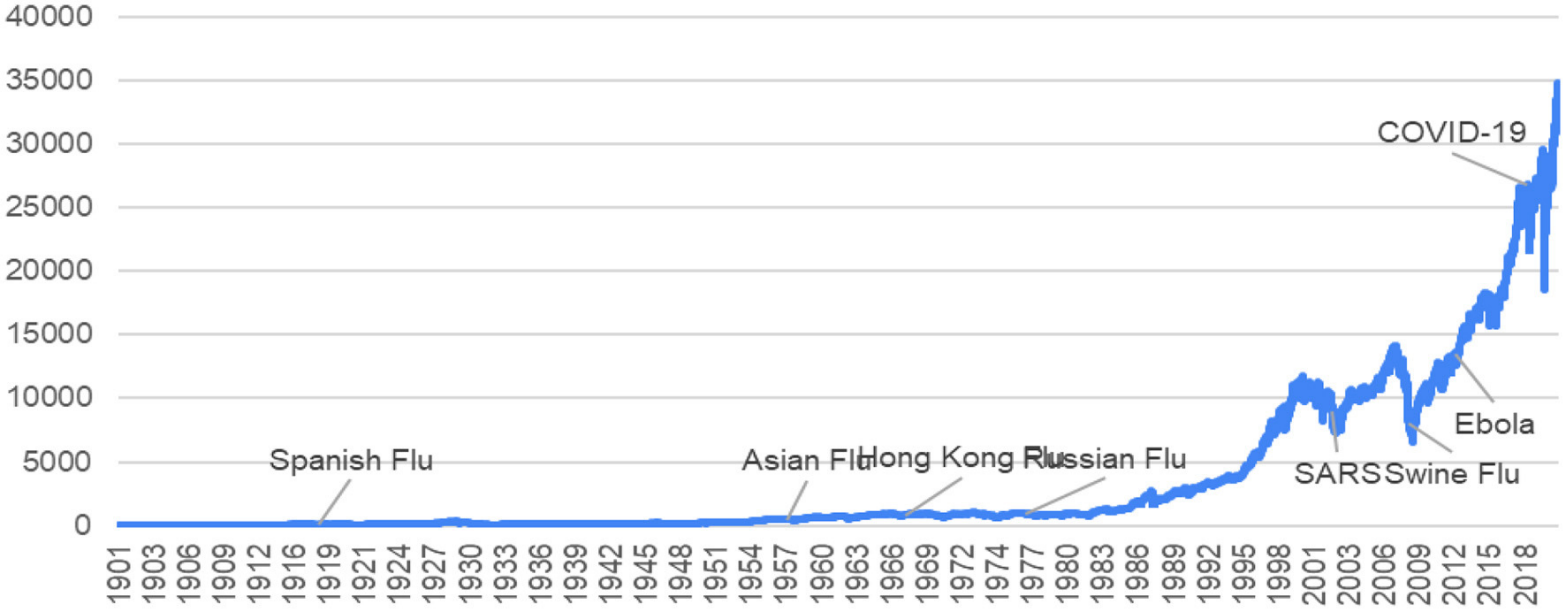
World pandemics and the movement of the Dow Jones Industrial Average (DJIA).

The effects of historical epidemics and pandemics on the financial market vary depending on location and era. Figure 1 presents the slip chart of the Dow Jones Industrial Average (DJIA) index and the historical pandemic records. Not all pandemics make the stock market go down a lot. The epidemics in the eighteenth and nineteenth centuries had limited financial effects because most epidemics were local ones that lasted a few months but not pandemics that affected the global world. In addition, different cities had their own stock exchanges, cross-market arbitrages stoped stock crashes in the infected cities. However, as human activities tend to globalization, worldwide pandemics show a different reality and then cause investor panic. Compared to the COVID-19 effects on the market with the Spanish Flu in 1918, the DJIA index stayed relatively stable, not dropping more than 5% during the period from 1918 to 1920. After it was over, the U.S. economy grew by 42% between 1921 and the stock market crash in 1929. The flu killed about 40 million people or 2% of the global population. About 550,000 Americans died of the flu, half a percent of the U.S. population. European and US stock markets reacted significantly, and negatively, to the surging death rates during the Spanish Flu (1). Since World War I was over at the end of 1918, so the overlap period makes it difficult to discriminate between the economic and financial effects of the war and those of pandemic. However, COVID-19 had a different effect on the U.S. stock market. The DJIA index was down 37% (on March 13) from an all-time high in February. At a similar time period, the S&P 500 and the tech-heavy Nasdaq dropped over 31 and 40%, respectively. The U.S. economy recovers gradually as job positions increasing and the service sectors reopening. Compared to literature focusing on the effect of the COVID-19 pandemic on the financial market (1–4), this paper attempts to find financial instruments in financial market for the public health care schemes (PHCS) to hedge pandemic risks.

There exist some catastrophe derivatives traded in financial market for hedging natural disasters such as catastrophe loss indexes, catastrophe bonds, and catastrophe equity puts or contingent surplus notes. A lot of literature study the effectiveness and efficience of catastrophe risk management via these derivative (5–7). Few financial derivatives are based on pandemics or epidemics. Less literature focus on how to employ financial instruments to manage pandemic risk. The World Bank issued two pandemic bonds with a total face value of US$320 million in 2017. These bonds were designed to offer funds to low-income countries to cover costs associated with the outbreak of any one of six diseases. Gründl and Regele (8) proposed a concept of pandemic partnership bonds, which governments could issue to insure themselves against future pandemic-related risks, with private and institutional investors providing the funding. This study shows that pandemic bonds may reduce the revenue volatility of public health care schemes.

The demand for medical treatments and vaccinations increases quickly as an epidemic becomes a pandemic. This tends to support the stock prices of firms engaged in medicine and biotechnology. If the relationship between newly diagnosed epidemics/pandemics and biotechnology stocks is strongly positive, insurers could build and adjust an investment portfolio of these stocks as a pandemic spread. The profit from the portfolio would offset some of the cost of pandemic-related claims. This study shows that these stocks are highly correlated to the spread of the COVID-19 pandemic.

This study makes three major contributions to the literature. First, we survey the statistical characteristics of pandemic historical records. The result shows that building a sound epidemic prevention system has become more necessary and important over time. Second, we show the feasibility of innovative financial instruments that could be employed effectively to cover a substantial amount of the cost of medical treatments borne by public health care schemes. Third, our empirical results show that the stock market is informationally efficient about the COVID-19 pandemic.

The remainder of this study is organized as follows. Section Analysis of Historical Pandemic Records analyzes the statistical characteristics of the pandemic historical records. Section A Hedge for Public Health Care Schemes develops the hedge instruments for public health care schemes. Section Empirical Results shows the empirical results, and the final section offers conclusions.

## Analysis of Historical Pandemic Records

As shown in Table 2, the recorded frequency of pandemics ranges from one to five per 100 years, with an average of three. The number of deaths per pandemic changed substantially over time, Regarding the pandemic frequency over the past 300 years, roughly corresponding to the post-Industrial Revolution period, the outbreaks per 100 years increase twice as large as the average in earlier times (from 2 to 4). In addition to the abovementioned serious pandemics, Jones et al. (9) collected 335 emerging infectious diseases (EIDs) from 1940 to 2004 and shows such diseases have risen significantly over time. They found that the origins of EIDs are significantly corrected with socio-economic, environmental, and ecological factors. Lindahl and Grace (10) also show EIDs are increasing, causing large losses in both human and animal lives, as well as huge costs to society locally and globally during the last few decades. Many factors contributing to EIDs include climate change, globalization, and urbanization, which are to some extent caused by humans after the post-Industrial Revolution period. Since the Wright brothers invented and flew the first airplane in 1903, people move more easily and quickly around the world than past due to advanced transportation, so will the virus accompanying humans. There were 4.2 billion air transport passengers in 2018—compared to 310 million in 1970. This mobility helped coronavirus' transfer from China in 2020 to more than 60 countries in 2 months. The chance of epidemic becoming pandemic becomes large. It means the pandemic can cause a lot of infected individuals and deaths across several geographic areas in a short term.

**Table 2:** Pandemics frequency and average deaths.

**Period**	**Frequency**	**Average deaths (million)**
1300–1400	1	50
1400–1500	1	0.017
1500–1600	4	18.5
1600–1700	1	Unknown
1700–1800	5	0.65
1800–1900	3	25.63
1900–2000	5	20.53
2000–2021	3	1.06

The average recorded deaths per pandemic were more than 20 million from 1800 to 2000 (largely due to the 1918 pandemic that killed at least 50 million people). While the number of pandemic-related deaths may seem to be increasing, a pioneer in infectious disease research, Robert Koch, who employed an optical microscope to observe the infectious pathogen Bacillus anthracis in 1875 that allowed scientists to learn more about the virus than ever before, proposed “Koch's Law” as a guideline to describe the pathogenesis of infectious diseases. The Law guides scientist on how to isolate the microorganisms from diseased individuals and grown in the experimental host to find the cure method.

The average number of recorded deaths for the most recent three pandemics are less obvious than those that occurred before 2000, as innovations in medical technology allow us to quickly find effective treatments and vaccines for new diseases. The establishment of modern public health care systems also offers more medical treatment for citizens than in the past. As a result, the number of pandemic-related deaths are lower now than in the past, although their frequency is increasing.

As a result of the industrial and agricultural revolutions, the standard of living in most countries has generally improved. At the same time, the world's population has increased, and the number of infected individuals naturally increases as the population grows. The United Nations estimated there were 7.9 billion people in the world at the end of 2020, and the number of confirmed cases of COVID-19 exceeded 138 million as of March 31, 2021. Although medical science has improved our ability to find treatments for pandemic diseases, the enormous cost of doing so creates another problem for the public health care system. A potential solution may be to employ new financial instruments to encourage private capital to help cover the large expenses caused by pandemics.

## A Hedge for Public Health Care Schemes

We define the gross inward premium P required to cover expected medical expenses L, and additional expenses s, as shown in Equation (1)

(1) $$\begin{array}{l} P=E\left[L\right]+s \end{array}$$

Net revenue *X* is simplified as premium income minus the total payoff for a claim *L*, expressed as

(2) $$\begin{array}{l} X=P-L \end{array}$$

The volatility of profits σ_*X*_ is equal to the volatility of losses σ_*L*_.

### A Pandemic Bond as a Liability Hedge

A public health scheme could issue *u* units of pandemic bonds to share the liability of pandemic-related medical expenses. Such bonds are similar to catastrophe bonds but are linked to pandemic-induced disasters. A pandemic bond is designed to have a principal (face value) *M* and an indemnity trigger point *K*, offering the contingent payment *H* = {*L* − *K* | (*M* + *K*) ≥ *L* ≥ *K*}, and charging a premium *p*_*H*_. The scheme would then obtain the contingent compensation:

(3) $$\begin{array}{l} L_{H}=uH \end{array}$$

The payment p_*H*_ made to bondholders would include the expected payoff *E*[*H*] plus a risk premium *C*_*H*_:

(4) $$\begin{array}{l} P_{H}=E\left[H\right]+C_{H} \end{array}$$

Net revenue with the hedge is expressed as follows:

(5) $$\begin{array}{l} X_{H}=X-uP_{H}+uH \end{array}$$

The volatility of revenues will decrease because the pandemic bond covers some or all the medical expenses that exceed the threshold *K*, and the payments made to bondholders decrease the scheme's revenue if medical expenses are below *K*.

The volatility of revenues σ_*X*_*H*__ for issuers of pandemic bonds is a function of *u*:

(6) $$\begin{array}{l} \sigma_{X_{H}}\left(u\right)=\sqrt{\sigma_{L}^{2}+u^{2}\sigma_{H}^{2}-2u\rho_{H}\sigma_{L}\sigma_{H}} \end{array}$$

where ρ_*H*_ denotes the correlation coefficient between *L* and *H*.

### The Biotechnology Index for Asset Hedge

A health care scheme may purchase *u* units of a biotechnology index (or individual biotech stocks) *I*, with the goal of realizing capital gains from that position when a pandemic occurs. The initial value is denoted as *uI*_0_. The value after the pandemic outbreak is *uI*.

The net revenue generated by the biotechnology index is as follows:

(7) $$\begin{array}{l} X_{I}=X-uI_{0}+uI \end{array}$$

The volatility σ_*X*_*I*__ of the position is a function of *u*:

(8) $$\begin{array}{l} \sigma_{X_{I}}\left(u\right)=\sqrt{\sigma_{L}^{2}+u^{2}\sigma_{I}^{2}-2u\rho_{I}\sigma_{L}\sigma_{I}} \end{array}$$

where ρ_*I*_ denotes the correlation coefficient between *L* and *I*.

Suppose that the risk management objective is to reduce the profit volatility to achieve the variance ratio $\frac{\sigma_{X_{I}}}{\sigma_{X}}$ (denoted by *d*), where 0 < *d* < 1. That is to say,

(9) $$\begin{array}{l} \sigma_{X_{I}}\left(u\right)-\left(1-d\right)\sigma_{L}^{2}\leq 0 \end{array}$$

In this case, the risk management objective above can be rewritten as

(10) $$\begin{array}{l} u^{2}-2\frac{\sigma_{L}}{\sigma_{X_{I}}}\rho u-\left(d^{2}-2d\right){\left(\frac{\sigma_{L}}{\sigma_{X_{I}}}\right)}^{2}\leq 0 \end{array}$$

This quadratic inequality holds if and only if it has real solutions, where

(11) $$\begin{array}{l} u_{1}=\frac{\sigma_{L}}{\sigma_{X_{I}}}\left(\rho -\sqrt{\rho^{2}-2d+d^{2}}\right)\text{ and}\\ u_{2}=\frac{\sigma_{L}}{\sigma_{X_{H}}}\left(\rho +\sqrt{\rho^{2}-2d+d^{2}}\right) \end{array}$$

In this case, the following inequalities must hold to ensure the existence of real roots

(12) $$\begin{array}{l} \rho >\sqrt{2d-d^{2}}\text{ or }\rho <-\sqrt{2d-d^{2}} \end{array}$$

Note that the absolute value of the correlation coefficient must exceed the threshold value of $\sqrt{2d-d^{2}}$ to achieve the risk management objective. This threshold increases with the ratio *d*, which means that a high correlation will reduce revenue volatility. If the correlation coefficient is positive, the scheme creates a long position of *u*_1_ units, because *u*_1_ #x0003C; *u*_2_ lowers hedging costs. Conversely, if the correlation coefficient is negative, the scheme creates a short position of–*u*_2_ units given that–*u*_2_ < –*u*_1_.

## Empirical Results

This section illustrates the relationship between the spread of COVID-19 and stocks related to medicine and biotechnology. As the pandemic's severity increased, the daily number of confirmed cases in the U.S. (denoted by ADCQC) increased. The stocks associated with epidemic prevention include medical treatments and vaccine development. For this study, we chose the following four indices, some represented by index-tracking ETFs that trade on U.S. stock exchanges, to characterize these types of assets: the NASDAQ Biotechnology Index (denoted by NBI), the NYSE Arca Biotechnology Index (denoted by BTK), the Dow Jones U.S. Health Care Index (denoted by DJUSHC), and the iShares U.S. Healthcare ETF (denoted by IYH). The data were obtained from January 2, 2020 to March 31, 2021, a total of 290 observations per item, from the Wind database in China.

**Figure 2:**
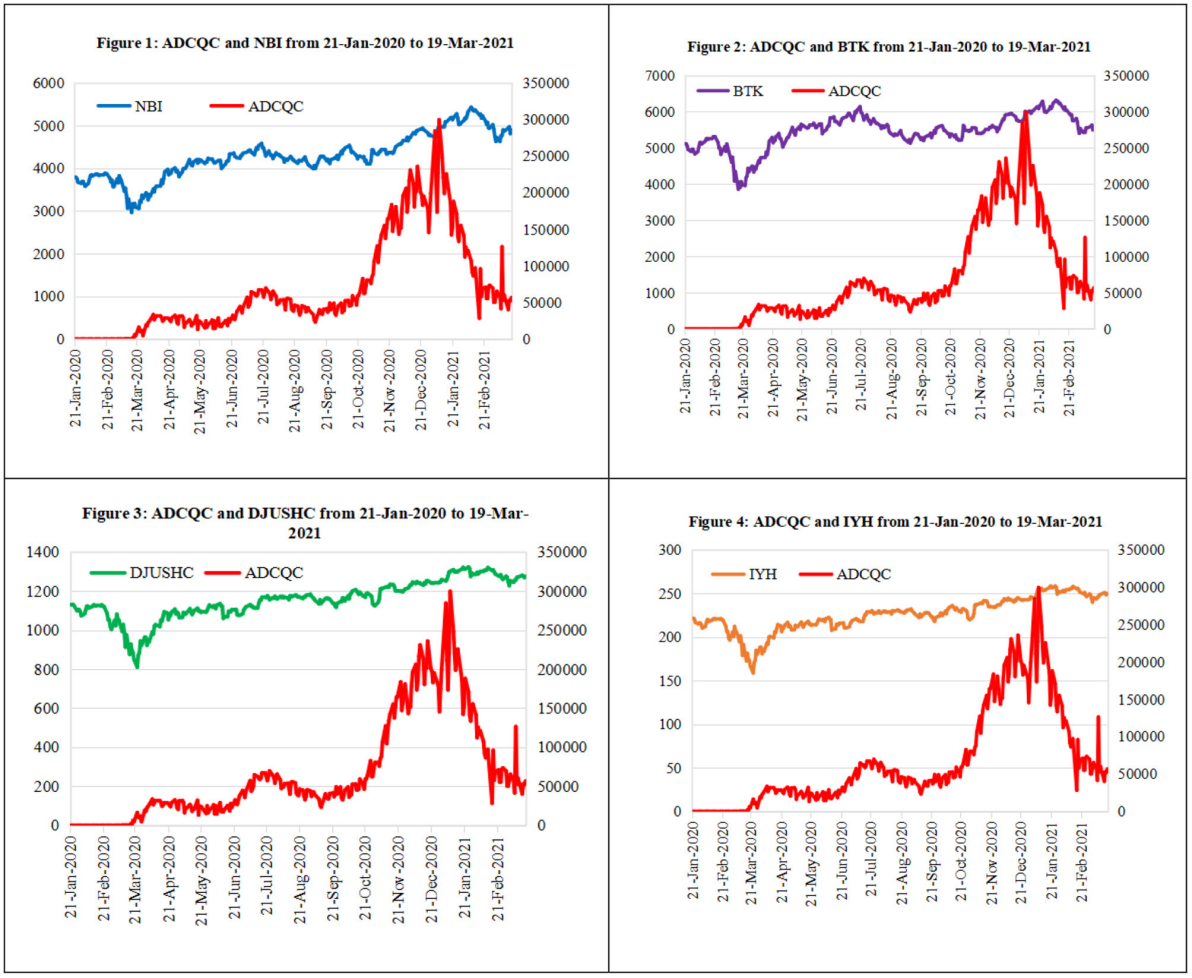
The trendlines of ADCQC, NBI, BTK, DJUSHC, and IYH.

Figure 2 shows that the levels of the four indices trended in the same direction as the ADCQC, indicating that the biotechnology and healthcare indices reflected the severity of the COVID-19 pandemic. Based on this, we continued with the analysis. Table 3 shows that changes in the NBI are approximately normally distributed, as its skewness and kurtosis are close to 0 and 3, respectively, and the Jarque–Bera test does not reject the null hypothesis. BTK, DJUSHC, and IYH are clearly negatively skewed and are leptokurtic, and their Jarque–Bera tests reject the hypothesis of a normal distribution. The correlation coefficients between ADCQC and the indices are >0.6, except for BTK (as shown in Table 4). Therefore, according to Equation (12), derivatives based on these underlying indices or ETFs could be used to hedge pandemic expenses.

**Table 3:** Descriptive statistics.

**Statistic**	**ADCQC**	**NBI**	**BTK**	**DJUSHC**	**IYH**
Mean	65118.52	4278.053	5444.884	1152.883	225.613
Maximum	299562	5427.13	6319.77	1322.44	258.36
Minimum	0	2961.94	3855.67	809.48	158.63
Std.Dev.	64488.41	513.989	469.677	98.753	19.232
Skewness	1.314	−0.025	−0.956	−0.62	−0.632
Kurtosis	3.880	2.803	4.303	3.603	3.616
Q (1%)	0	3062.5	3952.22	871.05	170.67
Q (10%)	2.5	3634.455	4889.415	1047.225	205.32
Q (25%)	23139	3986.13	5253.5	1098.61	214.98
Q (50%)	43834.5	4258.785	5497.02	1159.42	227.17
Q (75%)	80384	4579.42	5745.29	1230.72	240.71
Q (90%)	178143.5	4996	6000.24	1283.835	250.92
Q (99%)	248089	5361.57	6287.3	1319.53	257.96
Jarque-Bera	92.81	0.498	64.73	22.99	23.89
Probability	0.000	0.780	0.000	0.000	0.000
Observations	290				

*ADCQC, The daily confirmed numbers of quarantined cases of the USA; NBI, NASDAQ Biotechnology Index; BTK, NYSE Arca Biotechnology Index; DJUSHC, Dow Jones U.S. Health Care Index; IYH, iShares U.S. Healthcare ETF*.

**Table 4:** Correlation matrix (The USA).

	**ADCQC**	**NBI**	**BTK**	**DJUSHC**	**IYH**
ADCQC	1.0000				
NBI	0.6873	1.0000			
BTK	0.5378	0.9054	1.0000		
DJUSHC	0.6832	0.9423	0.8446	1.0000	
IYH	0.6797	0.9418	0.8454	0.9998	1.0000

The results of augmented Dickey–Fuller, Phillips–Perron, and Kwiatkowski–Phillips–Schmidt–ShinK tests shown in Table 5 show that their first differences reject the presence of a unit root. Furthermore, results of the Engle–Granger test shown in Table 6 indicate the existence of cointegration between ADCQC, NBI, DJUSHC, and IYH, but not BTK. Therefore, in our regressions, we assume that *X* = ADCQC and *Y* = (NBI, DJUSHC, IYH). First, we regress *Y* on *X* as follows:

(13) $$\begin{array}{l} Y=b+X, \end{array}$$

where *b* is an intercept term. The empirical results in Table 7 show that the coefficients of ADCQC are significantly positive, indicating that ADCQC leads to changes in NBI, DJUSHC, and IYH.

**Table 5:** Unit root test results.

	**Level**			**First differences**		
	**ADF**	**PP**	**KPSS**	**ADF**	**PP**	**KPSS**
ADCQC	−1.903	−1.750	1.53***	−17.891***	−25.063***	0.142
NBI	−0.997	−1.141	2.02***	−11.670***	−20.542***	0.0503
BTK	−1.600	−1.871	1.2***	−11.304***	−20.132***	0.0602
DJUSHC	−0.942	−1.220	1.98***	−11.162***	−21.560***	0.0907
IYH	−0.947	−1.227	1.97***	−11.093***	−21.353***	0.0897

***, **, and *indicate significance at the 1, 5, and 10% levels, separately.

**Table 6:** Engle-Granger cointegration tests results.

	**NBI**	**BTK**	**DJUSHC**	**IYH**
ADCQC	−3.273*	−2.977	−3.486**	−3.464**
Observations (1st step)	290	290	290	290
Observations (test)	289	289	289	289

***, **, and * indicate significance at the 1, 5, and 10 % levels, separately.

**Table 7:** OLS regression analysis results.

	**(1)**	**(2)**	**(3)**
	**NBI**	**DJUSHC**	**IYH**
Intercept	3921.349*** (31.23898)	1084.757*** (6.033766)	212.4136*** (1.18033)
ADCQC	0.0054778*** (0.0003412)	0.0010462*** (0.0000659)	0.0002027*** (0.0000129)
Observations	290	290	290
R-squared	0.4723	0.4667	0.4620
Adj R-squared	0.4705	0.4649	0.4601

***, **, and *indicate significance at the 1, 5, and 10 % levels, separately. ADCQC is the independent variable. The dependent variables include NBI, DJUSHC, and IYH.

Second, we regress X on Y to determine whether NBI, DJUSHC, and IYH can reflect the pandemic severity in advance.

(14) $$\begin{array}{l} X=c+Y, \end{array}$$

where *c* is an intercept term. The results provided in Table 8 show that the coefficients for NBI, DJUSHC, and IVH are significantly positive, indicating that the U.S. stock market exhibits informational efficiency about the COVID-19 pandemic.

**Table 8:** OLS regression analysis results.

	**(1)**	**(2)**	**(3)**
	**NBI**	**DJUSHC**	**IYH**
Intercept	−303778.5*** (23139.43)	−449227.3*** (32513.75)	−449067.3*** (32817.34)
ADCQC	86.23012*** (5.37038)	446.1388*** (28.09958)	2279.064*** (144.9349)
Observations	290	290	290
R-squared	0.4723	0.4667	0.4620
Adj R-squared	0.4705	0.4649	0.4601

***, **, and * indicate significance at the 1, 5, and 10 % levels, separately. The independent variables are NBI, DJUSHC, and IYH. ADCQC is a dependent variable.

## Conclusions

Trends from globalization, urbanization, and climate change are fueling the increased incidence of pandemic outbreaks (9, 10). The pandemics causing large losses in both human and animal lives have challenged public health care schemes in many countries. This study analyzes statistical characteristics of pandemics using historical records since the Middle Ages (11) and found that over the past 300 years, roughly corresponding to the post-Industrial Revolution period, the pandemic outbreaks per one 100 years increase twice as large as the average in earlier times (from 2 to 4). The average number of recorded deaths per pandemic was more than 20 million from 1800 to 2000. The average recorded deaths in the recent three pandemics are less obvious than those since 2000. So, the pandemic deaths are not greater than those in previous periods, although the frequency has increased. The contributions come from both innovations in medical technology and the establishment of a modern public health care system.

Compared to literature focusing on the effect of the COVID-19 pandemic on the financial market (1–4), this paper aims to find financial instruments for the public health care schemes (PHCS) to hedge pandemic risks. During the pandemic period, the PHCS bear a large number of medical expenses. A biotechnology investment portfolio assets can help the PHCS to increase their assets. Pandemic bonds can contribute to reducing liabilities. Empirical results indicate that such financial instruments may be practical, as the NASDAQ Biotechnology Index, Dow Jones U.S. Health Care Index, and iShares U.S. Healthcare ETF have positively correlated with the daily numbers of COVID-19 confirmed cases in the U.S. In addition, we found that the U.S. stock market shows informational efficiency with respect to the COVID-19 pandemic.

## Data Availability Statement

The raw data supporting the conclusions of this article will be made available by the authors, without undue reservation.

## Author Contributions

ZL: supervision, conceptualization, and resources. Y-CW: conceptualization, methodology, and writing—original draft, reviewing and editing. M-CW: methodology and writing-original draft preparation. YD: data curation and formal analysis. T-TN: software, visualization and editing. All authors contributed to the article and approved the submitted version.

## Conflict of Interest

The authors declare that the research was conducted in the absence of any commercial or financial relationships that could be construed as a potential conflict of interest.

## Publisher's Note

All claims expressed in this article are solely those of the authors and do not necessarily represent those of their affiliated organizations, or those of the publisher, the editors and the reviewers. Any product that may be evaluated in this article, or claim that may be made by its manufacturer, is not guaranteed or endorsed by the publisher.

## References

[1] Burdekin RCK. Death and the stock market: international evidence from the Spanish flu. Appl Econ Lett. (2020) 1–9. 10.1080/13504851.2020.1828802

[2] Bai L Wei Y Wei G Li X Zhang S. Infectious disease pandemic and permanent volatility of international stock markets: a long-term perspective. Fin Res Lett. (2021) 40:101709. 10.1016/j.frl.2020.101709 32837383 PMC7391063

[3] Chang CP Feng GF Zheng M. Government fighting pandemic, stock market return, and COVID-19 virus outbreak. Emerg Mark Fin Trade. (2021) 57:1–18. 10.1080/1540496X.2021.1873129

[4] Zhang D Hu M Ji Q. Financial markets under the global pandemic of COVID-19. Fin Res Lett. (2020) 36:101528. 10.1016/j.frl.2020.101528 32837360 PMC7160643

[5] Wang JY Wu WL Wu YC Yang MJ. How to manage long-term financial self-sufficiency of a natural catastrophe insurance fund? The feasibility of three bailout programmes. Eur Fin Manage. (2017) 23:951–74. 10.1111/eufm.12111

[6] Wu YC. Reexamining the feasibility of diversification and transfer instruments on smoothing catastrophe risk. Insurance Math Econ. (2015) 64:54–66. 10.1016/j.insmatheco

[7] Wu YC Yang MJ. The effectiveness of asset, liability, and equity hedging against the catastrophe risk: the cases of winter storms in north America and Europe. Eur Fin Manage. (2018) 24:893–918. 10.1111/eufm.12143

[8] Gründl H Regele F. Pandemic Insurance Through Pandemic Partnership Bonds. A Fully Funded Insurance Solution in a Public Private Partnership (No. 86). SAFE Policy Letter (2020). Available online at: http://nbn-resolving.de/urn:nbn:de:hebis:30:3-534499

[9] Jones KE Patel NG Levy MA Storeygard A. Balk D Gittleman JL. Global trends in emerging infectious diseases. Nature. (2008) 451:990–3. 10.1038/nature06536 18288193 PMC5960580

[10] Lindahl JF Grace D. The consequences of human actions on risks for infectious diseases: a review. Infect Ecol Epidemiol. (2015) 5:30048. 10.3402/iee.v5.30048 26615822 PMC4663196

[11] Pompella M Scordis NA. The Palgrave handbook of unconventional risk transfer. In: Pompella M Scordis NA editors. The Palgrave Handbook of Unconventional Risk Transfer. New York, NY: Springer International Publishing (2017). p. 467–71. 10.1007/978-3-319-59297-8

